# Use of a novel two‐dimensional ionization chamber array for pencil beam scanning proton therapy beam quality assurance

**DOI:** 10.1120/jacmp.v16i3.5323

**Published:** 2015-05-08

**Authors:** Liyong Lin, Minglei Kang, Timothy D. Solberg, Thierry Mertens, Christian Baumer, Christopher G. Ainsley, James E. McDonough

**Affiliations:** ^1^ Department of Radiation Oncology University of Pennsylvania Philadelphia PA USA; ^2^ IBA Dosimetry Schwarzenbruck Germany; ^3^ Westdeutsches Protonentherapiezentrum Essen Germany

**Keywords:** proton therapy, PBS, quality assurance, recombination

## Abstract

The need to accurately and efficiently verify both output and dose profiles creates significant challenges in quality assurance of pencil beam scanning (PBS) proton delivery. A system for PBS QA has been developed that combines a new two‐dimensional ionization chamber array in a waterproof housing that is scanned in a water phantom. The MatriXX PT has the same detector array arrangement as the standard ${\text{MatriXX}}^{\text{Evolution}}$ but utilizes a smaller 2 mm plate spacing instead of 5 mm. Because the bias voltage of the MatriXX PT and Evolution cannot be changed, PPC40 and FC65‐G ionization chambers were used to assess recombination effects. The PPC40 is a parallel plate chamber with an electrode spacing of 2 mm, while the FC65‐G is a Farmer chamber FC65‐G with an electrode spacing of 2.8 mm. Three bias voltages (500, 200, and 100 V) were used for both detectors to determine which radiation type (continuous, pulse or pulse‐scanned beam) could closely estimate $P_{\text{ion}}$ from the ratios of charges collected. In comparison with the ${\text{MatriXX}}^{\text{Evolution}}$, a significant improvement in measurement of absolute dose with the MatriXX PT was observed. While dose uncertainty of the ${\text{MatriXX}}^{\text{Evolution}}$ can be up to 4%, it is <1% for the MatriXX PT. Therefore the ${\text{MatriXX}}^{\text{Evolution}}$ should not be used for QA of PBS for conditions in which ion recombination is not negligible. Farmer chambers should be used with caution for measuring the absolute dose of PBS beams, as the uncertainty of $P_{\text{ion}}$ can be <1%; chambers with an electrode spacing of 2 mm or smaller are recommended.

PACS number: 87.53.Qc

## I. INTRODUCTION

Measurement of output and dose profiles at multiple depths is required for regular machines and patient‐specific quality assurance (QA). The need to accurately and efficiently verify output and profiles creates significant challenges in quality assurance of pencil beam scanning (PBS) proton beams. IBA Dosimetry (Schwarzenbruck, Germany) has developed a system called DigiPhant that combines a new two‐dimensional ionization chamber array dedicated to address the high dose rates in PBS, called MatriXX PT, in a waterproof housing that can be scanned in a water phantom. Use of the ${\text{MatriXX}}^{\text{Evolution}}$, an earlier version of the MatriXX PT, has been described by Arjomandy et al. for both periodic proton machine^(1)^ and patient^(2)^ QA, in combination with a plastic water phantom. In addition to providing improved measurement accuracy, the MatriXX PT easily facilitates measurements over multiple depths required for PBS machine and patient QA.

**Figure 1:**
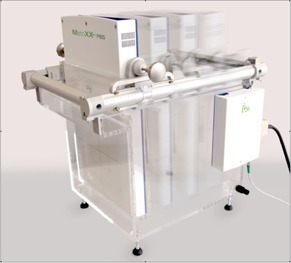
Setup picture of DigiPhant phantom with beam coming from the left direction and the detector array moving in four locations within the water tank.

Commissioning of dose computation models for spot scanning proton beams and treatment planning for PBS delivery have been described.^3^, ^4^ Current treatment planning systems (TPS) have deficiencies in the calculation of dose distribution due to inaccurate modeling of beam halo from the treatment nozzle and phantom material.^5^, ^6^, ^7^ Ion recombination also varies with beam and measurement conditions and detector design.^8^, ^9^ Because of this, we focus on the magnitude of improvement provided by the DigiPhant system (shown in Fig. 1) over methods using conventional chamber arrays and solid phantoms, with a particular emphasis on absolute dose measurement.

## II. MATERIALS AND METHODS

The MatriXX PT utilizes 1020 vented parallel plate chambers with a 7.6 mm center‐to‐center spacing between chambers, within the plane that is perpendicular to beam direction, in the same 32×32 arrangement as the ${\text{MatriXX}}^{\text{Evolution}}$, with the outermost four corner chambers removed. In contrast to the ${\text{MatriXX}}^{\text{Evolution}}$, however, the plate spacing is significantly smaller, 2 mm compared to 5 mm. For a given bias voltage of 500 V, smaller electrode spacing reduces ion recombination.^8^, ^9^ In addition, the MatriXX PT utilizes a 6 mm thick ABS (acrylonitrile butadiene styrene) buildup on top of detector array in contrast to the 3 mm buildup for the ${\text{MatriXX}}^{\text{Evolution}}$. This ensures a smooth manufacturing and assembly process supporting mechanical stability.

To align the system, the DigiPhant has two parallel side marks on both sides of one wall of the water tank. Reference marker on the MatriXX PT are then aligned to the parallel markers on the tank wall, providing a geometric distance and water‐equivalent thickness (WET) between the effective point of measurement and the tank surface of 100 mm and 102.6 mm, respectively. The water tank and MatriXX PT detector housing are composed of PMMA (acrylic) and are 10 mm and 5 mm thick, respectively. Because of tank wall, PMMA housing, and detector buildup, the most proximal depth the DigiPhant is capable of is 27 mm, while the most distal depth is 337 mm. To verify the vendor‐supplied WET calculation, the depth dose of a spread out Bragg peaks (SOBP) in double‐scattering (DS) treatment was measured with a multilayer ionization chamber array Zebra system and the DigiPhant system. The principle of multilayer ionization chamber array was described by Nichiporov et al.^(10)^ and the specific Zebra system (IBA dosimetry) by Dhanesar et al.^(11)^ Software for DigiPhant measurements allow acquisition of two‐dimensional profiles which can be converted to absolute dose by a user‐defined calibration factor. The calibration factor is acquired by averaging over the central 2×2 detectors in a uniform field with known dose. The OmniPro I'm*RT* software program is used for analysis of DigiPhant data.

Absolute output was measured using the MatriXX PT and ${\text{MatriXX}}^{\text{Evolution}}$ for several PBS proton beam energies and measurement depths. A region of interest covering a $1\times 1\;{\text{cm}}^{2}$ area, with dose mainly from the central 2×2 ion chamber array elements, was used for the absolute dose measurement. The same physical thickness of water or Solid Water (10 and 20 cm) was placed in front of the detectors to study the water equivalent property of Solid Water phantoms (Gammex Inc., Middleton, WI). The water‐equivalent thickness (WET) of each Solid Water phantom was experimentally determined, with WET ratios between 1.025 and 1.03.^(12)^ To avoid the potential output difference due to WET uncertainty, PBS beams were scanned with a mixture of energies to achieve a similar SOBP dose distribution as in double scattering (DS) delivery. Three such beams were used to achieve a uniform dose between depths of 20 cm and 12 cm with a modulation of 8 cm (R20M8), between depths of 12 cm and 8 cm with a modulation of 4 cm (R12M4), and between depths of 32 cm and 22 cm with a modulation of 10 cm (R32M10). A spot spacing of 0.5 cm and field size of 10 cm was used for R20M8, and a spot spacing of 0.4 cm and field sizes of 10 cm and 20 cm (F10 and F20) were used for R12M4 and R32M10 beams. To study the impact of spot size and energy on the ionization recombination, measurement depths were varied from 5 cm to 19 cm of R20M8 beam.

Ionization recombination, $P_{\text{ion}}$, is typically characterized using the ratio of collected charge at a nominal voltage to that at a lower voltage. Depending on the selected radiation type (continuous, pulse or pulse scanned), the ratio of collected charge is converted differently to $P_{\text{ion}}$.^13^, ^14^ Boutillon,^(15)^ Zankowski and Podgorsak,^(16)^ and Palmans et al.^(17)^ have reported that the use of pulse and pulse scanned beam overestimates $P_{\text{ion}}$, while a continuous beam underestimates $P_{\text{ion}}$. The bias voltage of the MatriXX PT and Evolution cannot be directly changed; therefore, ionization chambers PPC40 and FC65‐G were used to estimate potential differences in recombination characteristics between the MatriXX PT and Evolution. The PPC40 is a parallel plate chamber with an electrode spacing of 2 mm; the FC65‐G is a Farmer chamber with an electrode spacing of 2.8 mm. Therefore, the PPC40 chamber exhibits the same recombination conditions as the MatriXX PT. Also the FC65‐G was used to compare absolute dose measurements with PPC40 based on the IAEA 398 protocol.^(14)^ To study which radiation type (continuous, pulse or pulse‐scanned beam) could closely estimate $P_{\text{ion}}$, two lower bias voltages of 200 and 100 V were used for both the PPC40 and FC65‐G detectors in addition to the standard operating bias voltage of 500 V. The MatriXX PT and Evolution were calibrated in‐house to agree with the absolute output reported by the PPC40 and FC65‐G.

Ion recombination changes with beam current, beam duration, and dose rate and, therefore, with measurement depth and location. Since varying prescription dose also indirectly impacts beam current, beam duration, and dose rate, we use dose nonlinearity in this work to estimate the magnitude of ion recombination within the clinical dose range. The original prescription was 1 Gy‐equivalent within the uniform dose regions.

## III. RESULTS

**Figure 2:**
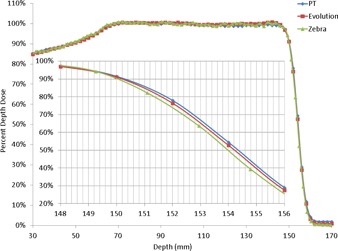
Comparison of proton beam depth dose curve in water obtained using a Zebra multilayer ionization chamber with that obtained using the MatriXX PT and Evolution in the DigiPhant water phantom. The buildup material thickness corrections of 6.2 mm and 3.1 mm were provided by IBA dosimetry.

Figure 2 shows the depth doses obtained using the Zebra and DigiPhant systems for a $10\times 10\;{\text{cm}}^{2}$ double scattering proton beam with a 10 cm SOBP. For the DigiPhant, the depth dose curves were measured every 2 mm to correspond with the inherent detector spacing of 2 mm in Zebra. The data measured with Zebra and DigiPhant systems were averaged over the 2.5 cm diameter and $1\times 1\;{\text{cm}}^{2}$ area, respectively. The WET of the PT and Evolution buildup material was determined to be 6.55 and 3.3 mm (using a distal range of 80%) and 6.5 and 3.3 mm (using a distal range of 90%), compared to the manufacturer specification of 6.2 and 3.1 mm, respectively. WET ratios of 1.159 for PMMA and 1.0375 for ABS were used in the WET calculation. The ∼0.3 mm WET difference between Zebra measurements and the vendor‐provided specification is within the 0.5 mm calibration accuracy of Zebra.^(11)^

**Figure 3:**
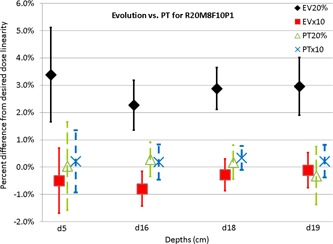
Percent difference of MatriXX PT and Evolution measured dose from the prescribed dose (10, 1, and 0.2 Gy equivalent at 16 cm depth) for depths of 5, 16, 18, and 19 cm of a pencil beam scanning beam that scans a cube with range of 20 cm, modulation of 8 cm, and field size of 10 cm.

Figure 3 shows the nonlinearity of dose measured at ten times and 20% of the prescription dose compared to that measured at the prescription dose. Up to 4% dose nonlinearity can be observed using the Evolution, but less than 1% using the PT. As the change in prescription dose indirectly affects the beam current, beam duration, and dose rate, the dose nonlinearity reflects the magnitude of ion recombination for most clinically relevant situations. The nonlinearity remains relatively constant with depth and shows the largest change between 20% and the nominal MU. The measurement uncertainty is greatest at the most proximal depth of 5 cm and 20% of the prescription dose, potentially due to smaller spots and lower signal. Due to differences in delivery systems (IBA PBS using a cyclotron versus Hitachi DS using a synchrotron), the dose nonlinearity of the ${\text{MatriXX}}^{\text{Evolution}}$ observed here is different from that reported by Arjomandy et al.^(1)^

**Figure 4:**
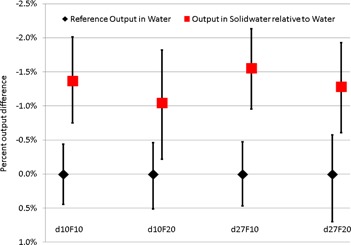
Output measured using the MatriXX PT in Solid Water relative to that in water, at depths of 10 and 27 cm, for PBS beams with a range of 12 cm and modulation of 4 cm, and a range of 32 cm and modulation of 10 cm, respectively. Scanning field sizes of 10 cm and 20 cm were used for both depths.

Figure 4 shows the difference in output in Solid Water and water measured using the PT in the center of uniform dose for a mixture of energies. Up to 1.5% dose difference was observed, though there was no strong dependence (1%–1.5%) between depths of 10 and 20 cm and field sizes of 10×10 or $20\times 20\;{\text{cm}}^{2}$ with measurement uncertainty of ∼0.5%. As dose is calculated in water for most TPS, the use of water in QA can reduce dose and WET uncertainties associated with Solid Water phantoms. Physical depths of 10 and 20 cm were used in water measurements. As the corresponding WET of 10.25 and 20.5 cm fall within the SOBP, there is no observable dose variation between the physical and WET depths. However, WET uncertainties of 0.5% between 1.025 and 1.03 (up to 1.5 mm error for 300 mm depth) can substantially impact dose profiles for complicated IMPT plans when the depth of interest is close to the distal falloff region rather than in the SOBP.^1^, ^2^, ^3^, ^4^

**Figure 5:**
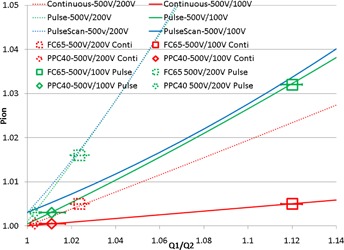
Ionization collection efficiency of Farmer chamber FC65‐G and parallel chamber PPC40 used to measure the absolute dose of MatriXX PT. Q1/Q2 is the ratio of collected charge under bias voltage of 500 V to that collected under bias voltages of 200 V or 100 V. Measurements are performed at the depth of 16 cm of a pencil beam scanning beam that scans a cube with range of 20 cm, modulation of 8 cm, and field size of 10 cm. Data points indicate $P_{\text{ion}}$ determined from the measured Q1/Q2 ratio using the continuous and pulse models. The curves (dash and solid curves) are calculated $P_{\text{ion}}$ from the charge ratio collected under bias voltages between 500 V and 200 V, and between 500 V and 100 V, based on the formula from Weinhous et al.,^(13)^ using continuous (red and shortened as C) and pulsed radiation types (green and shortened as P).

Figure 5 shows the charge collected at a depth of 16 cm for the same beam as Fig. 3 using PPC40 and FC65‐G chambers at bias voltages of 500, 200, and 100 V. The Q1/Q2 charge ratios between different bias voltages were converted to different $P_{\text{ion}}$ based on quadratic formula fit and the choice of radiation type as continuous or pulse beam. The $P_{\text{ion}}$ quadratic formula fit based on the beam model has a 0.3% difference from measurement when Q1/Q2=1.^13^, ^18^ Clearly the continuous beam type is the appropriate selection for the radiation type as pulse beam type would predict different $P_{\text{ion}}$ by 1.6% using charge ratio at 500 V over 200 V and 500 V/100 V. Similarly pulse scan beam type would predict different $P_{\text{ion}}$ by 1.7% (not shown in Fig. 5 for the sake of simplicity). Improper selection of pulse or pulse‐scanned beam type, as in the IAEA TRS 398, would result in an overestimate of $P_{\text{ion}}$ by <1.5% for the FC65‐G, but only <0.5% for the PPC40. In contrast, using the continuous beam type, we observed that the output reported by the FC65‐G is ∼0.2% lower than that reported by the PPC40. Because of choice of radiation type and the uncertainty of the initial, general recombination and charge multiplication terms in the continuous beam model, similar disagreements have been reported by Boutillon,^(15)^ Zankowski and Podgorsak,^(16)^ Palmans et al.,^(17)^ and Lorin et al.^(18)^ As a further consolidation of these observations, it should be mentioned that the beam duration of each spot is controlled to be longer than 3 ms. This interval is much longer than the typical ion drift collection time for such chambers.^8^, ^9^, ^13^, ^14^, ^15^, ^16^, ^17^, ^18^ As a consequence, the choice of the continuous beam model is appropriate, and the PPC40 should be recommended for calibration of absolute output as opposed to the FC65‐G.

## IV. DISCUSSION

In the case of IBA PBS,^(7)^ the beamline can have instantaneous dose rates that are higher than the maximum continuous dose rate of 26 Gy per minute reported by Palman et al.^(17)^ and could potentially cause the monitor chamber to exhibit ion recombination, as pointed out by Lorin et al.^(18)^ To ensure minimal ion recombination in the monitor chamber, Courtois et al.^(19)^ optimized the design and the bias voltage of the monitor chamber used in the IBA PBS systems, under various combinations of proton energies and beam currents evaluated, to achieve ion recombination less than 1% for all the instantaneous dose rates. Minimal ion recombination in the monitor chamber is consistent with our observation that the MU nonlinearity measured using the ${\text{MatriXX}}^{\text{Evolution}}$ is up to 4% but within 1% measured using the MatriXX PT for the same monitor chamber of the same beamline and measurement conditions. Clinical physicists are advised to work with their beamline vendors to perform similar experiments as Courtois and colleagues if over 1% nonlinearity is observed with the MatriXX PT.

The DigiPhant system is somewhat bulky, with a dimension of 540 mm (length) by 400 mm (width) and weight of 21 kg (empty) and 52 kg (filled with water); the MatiXX PT weighs 11 kg. This design is too heavy to be lifted. Further, it can accept beam angles of 90° or 270° only. Although it has smaller detection volume, in PBS the MatriXX PT has equivalent or better noise characteristics to the Evolution due to the lower ion recombination (∼1% error bars of Fig. 3).

## V. CONCLUSIONS

The DigiPhant system combines a new two‐dimensional ionization chamber array, the MatriXX PT, dedicated to PBS applications, within a waterproof housing that can be scanned a water phantom. Such a system is recommended for performing system and patient QA of proton PBS treatments to improve the absolute dose accuracy. As the dose uncertainty of MatrixX PT is <1%, but can be up to 4% for ${\text{MatriXX}}^{\text{Evolution}}$, ${\text{MatriXX}}^{\text{Evolution}}$ should not be used in PBS QA with high dose rates. Using a Farmer chamber for measuring the absolute dose of PBS beams should be performed with caution due to the uncertainty in $P_{\text{ion}}$. Chambers with electrode spacing of 2 mm or smaller should be recommended instead. Finally, it is important to note that the ${\text{MatriXX}}^{\text{Evolution}}$ is not electrically suited to be used in the DigiPhant.

## ACKNOWLEDGMENTS

Liyong Lin would like to acknowledge Bijan Arjomandy for the productive discussion regarding the studies of ion recombination.
